# Anorexia, Oral Health and Antioxidant Salivary System: A Clinical Study on Adult Female Subjects

**DOI:** 10.3390/dj7020060

**Published:** 2019-06-01

**Authors:** Marco Mascitti, Erminia Coccia, Arianna Vignini, Luca Aquilanti, Andrea Santarelli, Eleonora Salvolini, Jacopo Sabbatinelli, Laura Mazzanti, Maurizio Procaccini, Giorgio Rappelli

**Affiliations:** Department of Clinical Specialistic and Dental Sciences, Polytechnic University of Marche, Via Tronto 10, 60126 Ancona, Italy; marcomascitti86@hotmail.it (M.M.); dcermi@virgilio.it (E.C.); lucaquilanti1993@gmail.com (L.A.); mdocuniv@libero.it (E.S.); edottor@libero.it (J.S.); cr.anpath@libero.it (L.M.); m.procaccini@staff.univpm.it (M.P.); g.rappelli@univpm.it (G.R.)

**Keywords:** anorexia nervosa, oxidative stress, reactive oxygen species, saliva, superoxide dismutase

## Abstract

The aim of this study was to compare the oral health status and salivary antioxidant system between patients diagnosed with anorexia nervosa (AN) and healthy controls. A total of 25 female AN patients and 25 matched healthy controls were enrolled. Clinical parameters and saliva samples were collected for each patient. Two questionnaires to investigate oral health and hygiene were administered. Superoxide Dismutase (SOD) activity and High Reactive Oxygen Species (hROS) were evaluated. Salivary concentration of SOD was significantly higher in subjects with AN compared with control group (1.010 ± 0.462 vs. 0.579 ± 0.296 U/mL; *p* = 0.0003). No significant differences between groups were identified for hROS (233.72 ± 88.27 vs. 199.49 ± 74.72; *p* = 0.15). Data from questionnaires indicated that, although most of the patients recognized the oral hygiene importance in maintaining a good oral health, more than half of them had poor oral hygiene. Altered biochemical composition of saliva in patients with AN could be interpreted as an effective defence mechanism against oxidative stress. Moreover, despite the discrepancy between clinical findings and perception of the oral health in AN population arose, the quality of life of these patients appears not to be significantly affected by their dental condition.

## 1. Introduction

Eating disorders (ED) are a group of psychopathological disorders affecting patient relationship with food and her/his own body, which manifests through distorted or chaotic eating behavior [1]. Currently, the American Psychiatric Association has classified these disorders in: Anorexia Nervosa (AN), Bulimia Nervosa (BN) and Eating Disorders Not Otherwise Specified (EDNOS) [2]. AN is characterized by food restriction leading to underweight, BN is defined by binge eating and inappropriate compensatory behaviors, such as self-induced vomiting, use of laxatives/diuretics, and excessive exercise, while EDNOS refers to those who do not meet all the criteria of the other two syndromes. ED now constitute a true social epidemic, to such an extent that in European countries, the AN and BN are the most common disease among adolescents [3].

All of these disorders are associated with a wide range of adverse psychological, physical, and social consequences. ED can cause a number of medical complications due to malnutrition, self-induced vomiting and drugs abuse. Different organs and apparatuses may be affected with degrees of impairment correlated to the severity and the duration of the disease. Some organs and systems, as bone tissue, liver, kidney, and dental enamel, may undergo definitive damage not reversible with weight recovery [4,5]. All these medical complications, in most severe cases, can lead to the death of the patient. Indeed, the mortality rate of these subjects is much higher than that expected for the population of similar age, ranging between 5.9% and 8.4% [6]. Also, oral manifestations of EDs depend on duration and frequency of dysfunctional behaviors, induction of vomiting, use of medications, diet and oral hygiene level of the patient [7]. Affecting both soft and hard tissues, they include a number of signs and symptoms involving perioral tissues, oral mucosa, teeth (such as dental erosion and dental caries), periodontium, salivary glands, and temporomandibular joint [8].

Clinical signs of mucosal alteration include epithelial atrophy, erosions and ulcerations of the oral mucosa, which are often recorded in patients with ED. At the beginning, these mucosal alterations involve direct damage to DNA and other cell components, generating High Reactive Oxygen Species (hROS), which could cause a cascade of biological events, such as imbalance of DNA methylation or altered expression of growth factors [9,10,11].

To neutralize hROS and to prevent irreversible damages to cellular components, the organism has a powerful antioxidant defence system, that can be divided in two parts: enzymatic and nonenzymatic [12,13]. Several studies showed that the salivary antioxidants have high specificity in relation to oral pathologies [14,15]. For this reason, exploration of saliva for oxidative stress markers that accurately reflect the redox status of the oral cavity in relation to local pathologies may be of clinical interest [16].

Enzymatic antioxidant defence system seems to have a primary role in counteracting oxidative stress. Superoxide dismutase (SOD) is a major antioxidant enzyme present in substantial and stable concentration in saliva, suggesting being a useful biomarker for salivary antioxidant system [17]. Furthermore, to the best of our knowledge, no previous studies have investigated salivary SOD concentration of patients with ED.

The aim of this study was to the compare oral health status and salivary antioxidant system between patients diagnosed with anorexia nervosa (AN) and healthy controls. The null hypothesis was that there were no differences between these two groups.

## 2. Materials and Methods

Twenty-five consecutive patients, who were undergoing psychiatric and/or medical outpatient treatment at two clinics for AN (“Residenza Palazzo Francisci”, Todi, Italy and “Il Nido delle Rondini”, Testico, Italy), were enrolled in the present study between October and December 2015. The AN diagnoses were conducted by an expert team according to the diagnostic and statistical manual of mental disorders-IV (DSM-IV) criteria. The study was performed in accordance with the principles of the Declaration of Helsinki as revised in 2013 and it was approved by the Local Review Board (USLUmbria 1, Perugia, Italy, 8 September 2015). A written, informed consent was obtained from all participants.

Inclusion criteria were: (a) diagnosis of AN from at least 1 year; (b) age above 18 years. Exclusion criteria were: (a) illiteracy; (b) mental retardation; (c) history of diseases known to interfere with eating behaviors: diabetes mellitus, thyroid disease, and loss of appetite related to cachexia syndrome (e.g., cancer, AIDS, renal failure, advanced liver disease, multiple sclerosis).

For oral examination and salivary analysis, a control group of 25 subjects with no previous history of ED, matched for sex and age, was selected from the standard recall patients at the Dental Clinic Polytechnic University of Marche, Italy. The selection was made from the consecutive list of patients who were to be given an appointment for a routine check-up.

### 2.1. Oral Examination

For all participants, individual sociodemographic data, as well as general and oral health were recorded. In addition, dietary habits, such as consumption and frequency of acidic beverages were documented too. Clinical examination of AN and control groups were performed by two trained dentists (E.C. and G.R.). All the patients were checked individually to evaluate the presence of caries, fillings, erosion, cervical or cusp defects, and dental mobility. The lingual/palatal and buccal surfaces of all teeth as well as the occlusal surface of premolars and molars were examined. For severity grading of dental erosion, the Basic Erosive Wear Examination (BEWE) scoring system was used (0 = No erosion; 1 = Initial erosion; 2 = Moderate erosion; 3 = Severe erosion) [18]. The number and distribution of affected teeth and surfaces were also registered. Plaque index, periodontal probing depth, clinical attachment level, Periodontal Screening and Recording Index (PSR) and presence of bleeding on probing were measured at 6 sites for each tooth, except third molars, and recorded. Periodontal status was assessed using the PSR. A specialized periodontal probe with a ball-shaped tip with a 0.5 mm diameter was used in order to examine and score six sites per tooth in each patient dentition sextant on a 0 to 4 hierarchical grading scale, with only the highest PSR score per sextant recorded. The index uses a common evaluation method based on the following three periodontal disease indicators: gingival bleeding on probing, calculus accumulation and probing depth. In addition, the PSR Index provides a more detailed picture of periodontal status by recording the presence of tooth mobility, furcation involvement, gingival recessions exceeding 3.5 mm and muco-gingival problems. When at least one of the above conditions is present, an asterisk is recorded with the PSR score for that given sextant [19]. The status of the salivary glands was assessed by visual examination and palpation. Temporomandibular joint disorders as well as the presence of xerostomia and cracked and dehydrated lips were investigated. Soft tissues lesions, DMFT, and degree of oral hygiene were recorded too. The examiner was blinded to the medical history and to the results of the questionnaires.

### 2.2. Questionnaires

Prior to the dental examination, the AN patients were interviewed by one examiner (A.V.). For this study, two questionnaires were used. The first was an “ad hoc” tool consisting in 10 items used to evaluate the oral hygiene habits of the individual patients. Oral hygiene habits, brushing teeth frequency, and the presence of oral disorders such as bleeding gums, dentinal hypersensitivity and sensation of dry mouth were investigated among others.

The second questionnaire was the “Oral Health Impact Profile-14” (OHIP-14), a tool designed to assess the quality of life in relation to oral health, investigating mainly psychological and behavioral problems [20]. All of the OHIP-14 items are understood as negative outcomes, and therefore the tool does not fit all the positive aspects of oral health. Each “item” includes a question related to a particular aspect of the perception of oral health status at which the subject can respond by choosing between 5 degrees of response, according to a qualitative ordinal scale, with a score from 0 to 4 (0 = Never; 1 = Almost never; 2 = Occasionally; 3 = Often; 4 = Very often). In this study, the “additive method” (calculation of the total score by adding the scores of each item) was used. Using this method, the sum of the responses produces a total score ranging from zero (best possible score) to 56 (worst possible score). Thus, the lower score corresponds to better perceived oral health; while the higher value obtained corresponds to a worse one.

### 2.3. Saliva Samples

Saliva samples were collected from each patient and the control group. All subjects were instructed to refrain from eating and drinking, as well as from oral hygiene and smoking, for 1h before the samples collection. Saliva sampling was performed by the standardized Salivette^®^ method following the instructions of the manufacturer [21], and it was programmed in such a way that there were no more than 2–3 patients a day. The samples of the collected saliva were centrifuged by two trained scientists (E.S. and J.S), and the recovered salivary fluid was aliquoted and stored at −80 °C until use. All these procedures took place within 1 h between sampling and processing of saliva samples.

SOD activity was evaluated using the DetectX^®^ Superoxide Dismutase (SOD) Colorimetric Activity Kit (K028-H1, Arbor Assays, Ann Arbor, MI, USA), following the manufacturer instructions instructions and previously reported [22]. Briefly, superoxide anion, generated from the conversion of xanthine and oxygen to uric acid and hydrogen peroxide by xanthine oxidase, converts water-soluble tetrazolium 1 (WST-1) salts to WST-1 formazan, a colored purple product that absorbs light at 450 nm. SOD reduces the superoxide ion concentration and thereby lowers the rate of WST-1 formazan formation. The extent of inhibition by SOD depends on the superoxide generation rate. Therefore, SOD activity is determined by the percent inhibition of the rate of WST-1 formazan production and expressed in U/mL.

The hROS levels were determined by the dye probe hydroxyphenyl fluorescein (Cell Technology Inc., Mountain View, CA, USA), which selectively detect hydroxyl radical and peroxynitrite, as already reported [22]. Who performed the salivary analysis was blinded to the clinical findings, medical history and to the results of the questionnaires.

### 2.4. Statistical Analysis

All data were analyzed using the software package IBM SPSS Statistics for Windows, Version 23.0 (IBM Corp., Armonk, NY, USA). The normal distribution of continuous variables was tested by the Kolmogorov–Smirnov test. Differences in personal data, periodontal status, and dental status between Patients and Control group were determined using t-student with Weich correction test. Differences in SOD activity and hROS expression between Patients and Control group, expressed in U/mL and RFU respectively, were determined using the Mann–Whitney U-test. The linear regression analysis was used to detect any possible correlations between the variables tested. Data were expressed as Mean ± SD. A value of *p* < 0.05 was considered statistically significant.

## 3. Results

Of the 25 AN subjects included in this study, all were female with a mean age of 24.5 ± 9.2 years (range 18–56). 56% of patients was taking antidepressant drugs, xerostomia and dysgeusia were daily reported by 20% of patients, while dental hypersensitivity was reported once a week by 16% of patients. Moreover, the 76% of patients never reported glossodynia or facial pain.

Results of oral cavity examination were summarized in Table 1. 76% of AN patients presented several dental erosions, showing a mean BEWE score of 5.24 ± 4.47. A total of 104 dental erosions were found: 75% were classified as initial erosion, 24% as moderate and 1% as severe. The dental surfaces most frequently involved were the occlusal surfaces of the molar and premolar regions of the mandible. DMFT mean value was 6.80 ± 3.76 (range 0–12): in particular, 40 dental caries were found in 14 patients (mean value of 1.60 ± 2.08; range 0–7), while mean value of missing teeth was 3.04 ± 2.05 (range 0–7). Regarding dental fillings, 16 patients presented 54 dental fillings (mean value of 2.16 ± 1.99; range 0–6). Significant differences regarding the number of missed and filled teeth, DMFT and BEWE scores were found between AN patients and control subjects (Table 2).

Questionnaire results showed that most of the patients give importance to oral hygiene, reporting to brush their teeth from 2 to 3 times a day for 2–3 min. Even if the 76% of the patients reported a correct brushing technique, 52% uses the toothbrush on all teeth surfaces. Moreover, 45% of the patients used dental floss, toothbrush, and mouthwash. A total of 68% of the patients reported gingival bleeding on brushing. The dentinal hypersensitivity is one of the most reported symptoms (60%); while half of the patients reported xerostomia on monthly or daily basis. Score values for OHIP-14 ranged from 0 to 46 with a mean score of 20.92 ± 12.35.

**Figure 1:**
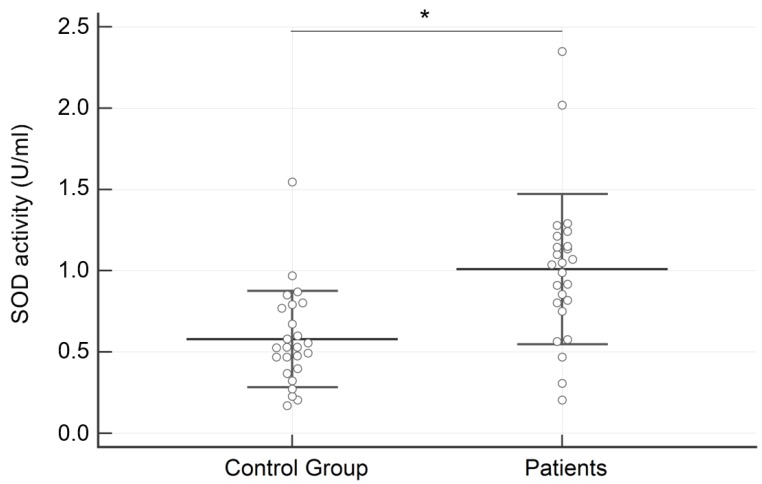
Results of the superoxide dismutase activity (SOD) expressed in U/mL in the two examined groups. Data are reported as Mean ± SD. * = *p* < 0.001.

**Figure 2:**
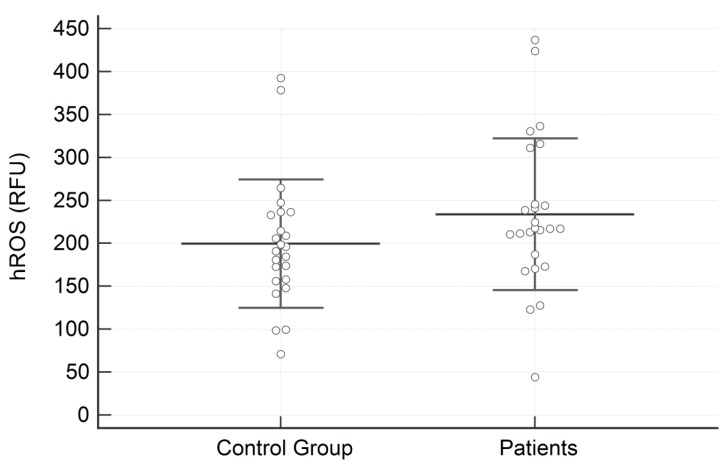
Results of the high reactive oxygen species (hROS) expressed in RFU in the two examined groups. Data are reported as Mean ± SD.

Regarding salivary analysis, the concentration of SOD was significantly higher in subjects with AN than in the control group (1.010 ± 0.462 vs. 0.579 ± 0.296 U/mL; *p* = 0.0003) (Figure 1). On the contrary, no significant differences between groups were identified for hROS (233.72 ± 88.27 vs. 199.49 ± 74.72 relative fluorescence units; *p* = 0.15) (Figure 2).

## 4. Discussion

The results of the present study indicated an alteration in biochemical composition of saliva in AN patients. Moreover, the obtained results could be interpreted as follows.

It has been reported that ED patients suffer of impaired oral health and a large number of oral symptoms. This is in agreement with the results of Johansson et al., who showed that self-reporting oral symptoms were two to three times higher in ED patients compared to the control group [23]. The difficulty in swallowing, at least more than once a month, was detected in 55% of subjects. It has been associated with depression, anxiety, somatization and psychological concerns, gastro-esophageal reflux or hyposalivation [24,25,26].

Taste alteration was reported by 45% of patients, in agreement with data reported by previous studies, where it was associated with mineral deficiency or unusual eating habits, binge eating and vomiting [27,28].

Except for atrophic glossitis or atrophic mucosa, no other soft tissue lesions were detected. The reduced supply of vitamins and other nutrients, as well as general metabolic abnormalities, iron deficiency, and anemia can strongly influence the biology of the oral mucosa determining a generalized atrophy. In particular, vitamin B group deficiency has been associated with a reduced regeneration of epithelial cells, mostly evident on the tongue. Mucosal atrophy may also cause a general feeling of oral burning, which may present more intense on the tongue, even if only 30% of the samples reported to have experienced glossodynia. Erythematous soft palate lesions of patients with purging behavior may be related to the action of acid direct contact during vomiting. Moreover, studies have shown that oral precancerous lesions such as lichen planus and leukoplakia, but also oral squamous cell carcinoma are associated with an increase in salivary markers of oxidative stress, such as malondialdehyde [29,30,31].

Dentine Hypersensitivityis one of the most reported symptoms (60%) and is probably due to the loss of dental tissue due to erosion, cervical defects and/or caries. There is agreement in confirming that dental erosion is a typical finding in ED patients [32,33]. Clinicians should be aware about the importance of a correct diagnosis of Dentine Hypersensitivity, excluding any confounding factors from other conditions that could lead to orofacial pain. Once the diagnosis of Dentine Hypersensitivity has been established, then the best treatment option should be suggested on the basis of its extension and severity (e.g., nerve desensitization, cover or plugging dental tubules, dentine sealers, periodontal soft tissue grafting, laser therapy, homeopathic medications) [33].

81% of subjects examined in the test group of this study presented erosive lesions. It has been reported that dental erosion does not appear until the regurgitation is not present for at least two years [34,35]. Conversely, Milosevic et al., found no linear relationship between the frequency and/or duration of the self-induced vomiting and dental erosion presence/severity [36]. Regarding the type of ED, it has been suggested that women with BN suffer from severe dental erosions and that subjects with self-induced vomiting or bulimic habits have a prevalence of dental erosions than patients with AN and EDNOS, whereas patients with AN are more likely to develop dental erosions compared to the healthy population [33]. Even Ohrn et al., support the fact that all individuals who suffer from any type of ED have a high risk of presenting dental erosion than the rest of the population and with a greater degree of severity [32]. Thus, the presence of numerous dental erosions should lead the clinician to suspect that the patient is suffering from an ED. In the present study, the teeth with more extensive erosions were the lower molars and premolars, followed by upper molars and premolars, the upper incisors and canines and finally the lower incisors and canines. In literature, upper incisors and canines are the teeth most affected by erosion; while those with the most severe erosion, are the occlusal surfaces of lower molars [37,38].

Since the etiology of teeth decay is multifactorial, the presence of caries cannot be entirely attributed to ED and their relationship is not conclusive [8,33,39]. Indeed, the susceptibility to caries in ED patients is not significantly higher from that of matched healthy individuals [40].

Hypertrophy of the parotid gland was reported to be one of the most common manifestation in patients with ED, with an incidence varying from 10% to 66% [23,32,41,42]. On the contrary, the patients evaluated in the present study had no signs of parotid swelling. However, salivary swelling was associated mostly with BN patients, while no one of the present sample suffered from BN.

Half of the patients reported xerostomia on monthly or daily basis. This may rely on antidepressant drug intake. Moreover, the association between xerostomia and poor oral hygiene may lead to plaque accumulation that in turn can promote tooth decay and/or periodontal inflammation. Gingivitis was observed in all the examined subjects, mostly associated with poor oral hygiene. The presence of dry lips, as a result of dehydration and self-induced vomiting, was detected in the majority of the patients (76.2%), in agreement with many reports [23]. However, this feature was not only present in patients suffering from ED and could also be due to other medical conditions.

When comparing oral hygiene status of ED patients with the results of self-reported questionnaires, a dichotomy between the clinical findings and the perception of the oral health in the studied population arose. Even if most of the patients recognized the oral hygiene importance in maintaining a good oral health and performed it in a satisfactory manner, more than half of the subjects showed a poor oral hygiene. Indeed, 70% presented gingival bleeding on probing and gum inflammation was detected in all of them. Even when looking at the OHIP-14 results, it seemed that the majority of subjects considered satisfactory their oral health. Therefore, the quality of life of these patients was not significantly affected by their dental condition.

As saliva reflects the current physiological and pathological body conditions [43,44], alterations in its biochemical composition could be interpreted as an indirect expression of the well-known metabolic impairments occurring in EDs. Moreover, the oxidative stress could play a role in the development and maintenance of AN or could be one of its downstream effects [45,46,47].

The identification of antioxidants in tissues, blood, saliva, and other body fluids provides an idea of the local or systemic defensive effectiveness of the individual, and saliva is considered an important defensive line against oxidative stress [48]. Among serum, plasma, whole blood and blood cell, saliva sampling represents an easy, non-expensive protocol, causing minimal discomfort [49].

The antioxidant salivary system is formed by some enzymes as SOD, which is mainly produced by parotid glands [48]. Previous proteomics studies showed that oral cavity express 3 isoforms of SOD: in particular, major salivary glands are sources of copper-zinc SOD, while minor salivary glands as sources of both copper-zinc and manganese SOD. Furthermore, the expression of extracellular SOD by submandibular and sublingual glands have been reported [50]. This enzyme is a marker of oxidative stress and it is capable of increasing in response to different inflammatory reactions, as has recently been confirmed by Bachmeier et al., [12], that demonstrated an increase in the concentration of SOD in processes such as tonsillitis, pulpitis, periodontitis and peri-implantitis. SOD in saliva is able to protect oral cavity against the negative effects of endogenous and exogenous ROS and it represents the first antioxidant defense in tissues [51,52].

In the present study, we measured the overall SOD activity, regardless of the specific isoform. Regarding hROS, their production mainly by neutrophils infiltrating periodontal tissue and gingival sulcus is well acknowledged.

Compared with their matched healthy controls, patients with ED differed significantly in salivary SOD concentration that was statistically significant increased. This significantly altered biochemical composition of saliva in patients with AN compared with healthy controls, observed in this study, could be interpreted as a defense mechanism of saliva against oxidative stress, probably due to atrophic mucosa, atrophic glossitis, gingivitis and immunosuppression induced by misfeeds. Indeed, the increase in the production of salivary SOD could be considered a partial compensatory response when there is oxidative stress in the oral cavity [52]. Thus, as described in the Bachmeier et al., the organism produces powerful anti-oxidant systems such as SOD in order to neutralize biological negative events caused by hROS [12]. High SOD levels found in AN patients could in turn partially neutralize the hROS values. In support of this hypothesis, no significant differences were found between the two groups in terms of hROS concentration.

Regardless of the periodontal status of AN patients, conflicting data are available in literature. For example, Romanos et al., reviewed the orofacial manifestations in patients with ED. According to these authors periodontal disease is an uncommon manifestation in EDs patients, suggesting that an avitaminosis-C may play a role in worsening the periodontal status of these subjects [53].

Tothóva et al., reported that oxidative stress has been implicated in the etiology and pathogenesis of periodontitis. In particular, the production of proteolytic enzymes and the respiratory burst of neutrophils mediated by enzymes such as NADPH oxidase and myeloperoxidase have been indicated as factors that lead to the generation of ROS and oxidative stress [43]. However, to the best of our knowledge, no previous studies have investigated the relationship between the periodontal status and salivary SOD concentration of AN subjects.

Almerich-Silla et al., attempted to determine the association between different periodontal bacteria and oxidative stress parameters [54]. Oxidative stress biomarkers, total antioxidant capacity, and antioxidant enzymatic activity were evaluated. A significant increase of all oxidative stress marker levels was observed, except for SOD concentration, suggesting the lack of an association between SOD and periodontal pathogens.

Novakovic et al., investigated the influence of non-surgical periodontal treatment on salivary antioxidants and evaluated their capacity as biomarkers in reflecting periodontal tissue condition and treatment outcome, showing a positive correlation between SOD and gingivitis [55]. SOD concentration was higher in patients with chronic periodontitis than in healthy control subjects; moreover, SOD concentration decreased in response to non-surgical periodontal treatment. On the contrary, Trivedi et al., assessing the activity of SOD levels in saliva of 30 patients diagnosed with chronic periodontitis compared to 30 healthy controls, reported that SOD levels were statistically significantly lower in the test group than in the control one [56].

Nevertheless, several confounding factors could have played a role in these results. First of all, although the subjects were instructed to refrain from drinking and smoking before the samples collection, it cannot be excluded that alcohol drinkers and long-term smokers could present altered basal levels of oxidative stress in saliva. Patients with gingivitis and periodontitis could have clinically imperceptible blood contamination of saliva, increasing SOD levels. Furthermore, the small sample size limits the statistical power of this study.

Alternatively, inter-individual variability in SOD levels could justify the apparent conflicting data available in literature. In particular, it is possible that host-related and external factors could influence the basal levels of SOD (e.g., periodontal treatment). Therefore, the findings of the present study could be interpreted as an effect of AN on periodontal health status.

## 5. Conclusions

In conclusion, the altered biochemical composition of saliva in patients with AN could be interpreted as an effective defense mechanism of saliva against oxidative stress. The increment in SOD concentration in AN patients could be interpreted as an effect of AN on oral health status. Moreover, even if a dichotomy between clinical findings and perception of the oral health in the studied population arose, the quality of life of these patients seems to be not significantly affected by their dental condition. However, due to the limited sample size, further studies on larger populations are necessary to confirm such hypotheses.

## Figures and Tables

**Table 1:** Summary of oral and dental manifestations in anorexia nervosa (AN) patients.

Clinical Feature	n. of Subjects (%)
***Periodontal status***	
- Gingivitis - Periodontal disease	23 (92.0) 2 (8.0)
***Dental status***	
- Dental erosions - Decayed - Missed - Filled	19 (76.0) 14 (56.0) 21 (84.0) 16 (64.0)
***Oral hygiene***	
- Poor - Good - Excelent	13 (52.0) 10 (40.0) 2 (8.0)
***Other disorders***	
- Atrophic glossitis - Atrophic mucosa - Temporomandibular joint disorders - Dry and chapped lips	9 (36.0) 4 (16.0) 7 (28.0) 17 (68.0)

**Table 2:** Comparison between AN patients and control group.

	AN Patients	Control Group	*p* Value
***Personal data***			
Age (mean ± SD)	24.5 ± 9.2	24.2 ± 5.4	0.8814 ^†^
Sex (n. female)	25	25	1.0000 ^†^
***Periodontal status (n.)***			
- Gingivitis - Periodontal disease	23	16	0.6378 *
	2	3	
***Dental status (mean ± SD)***			
- D - M - F - DMFT - BEWE	1.6 ± 2.1	2.5 ± 1.5	0.0812 ^†^
	3.0 ± 2.1	0.8 ± 0.7	<0.05 ^†^
	2.2 ± 2.0	1.0 ± 0.8	<0.05 ^†^
	6.8 ± 3.8	4.3 ± 2.2	<0.05 ^†^
	5.2 ± 4.5	0.6 ± 0.7	<0.05 ^†^

^†^*t*-test. * Fisher’s exact test.
